# Follow-Up Patterns at a Low-Threshold Mobile Medical Unit Providing Opioid Use Disorder Care in an Urban Setting: A Group-Based Trajectory Modeling Approach

**DOI:** 10.21203/rs.3.rs-9173540/v1

**Published:** 2026-04-14

**Authors:** Kanya K. Shah, Abigail T. Elmes-Patel, Sarah Messmer, Aaron Winn, Angela Kong, Lisa K. Sharp, Daniel R. Touchette

**Affiliations:** University of Illinois at Chicago; University of Illinois at Chicago; University of Illinois at Chicago; University of Illinois at Chicago; University of Illinois at Chicago; University of Illinois at Chicago; University of Illinois at Chicago

**Keywords:** Opioid Use Disorder, Low-Threshold Approaches, Mobile Medical Units, Group-Based Trajectory Modeling, Follow-Up Patterns, Buprenorphine Access

## Abstract

**Background:**

The University of Illinois Chicago (UIC) Community Outreach Intervention Project (COIP) established a low-threshold mobile medical unit that dispensed medications to treat opioid use disorder (MOUD) to individuals with high need. Knowledge of mobile unit utilization is limited to binary retention metrics. This study identified and characterized patterns of patient follow-up at a mobile unit dispensing MOUD.

**Methods:**

Group-based trajectory modeling (GBTM) identified patterns of follow-up observed at the mobile unit. The data source was an internal database of every UIC COIP mobile unit patient encounter, maintained by clinicians. Individuals visiting the mobile unit for MOUD services while the UIC mobile unit carried buprenorphine onsite were included. In the GBTM, follow-up data for each patient over 12 months after their first visit were aggregated to yield groups of individuals with similar follow-up patterns. After GBTM, a multinomial logistic regression was used to compare demographic, substance use, and treatment characteristics between identified follow-up pattern groups.

**Results:**

Of the 964 eligible individuals, 429 had more than one mobile unit visit and were included in the GBTM. The best fitting GBTM model had four-groups, and the resulting follow-up patterns were labeled based on trajectory shape. Adding a group of individuals who only visited the mobile unit once in the one-year observation period yielded five total groups of mobile unit utilizers: slow-decline (n, %: 53, 6%), fast-decline (159, 16%), infrequent (175, 18%), continued use (42, 4%), and single-visit (535, 56%). Characteristics within the five groups were generally similar, with the continued use group having the highest average number of visits in the one-year period. The slow-decline group had the highest rates of insurance and of receiving buprenorphine during their visit.

**Discussion/Conclusion:**

This study identified five follow-up patterns at a mobile unit providing MOUD to neighborhoods with unmet needs: slow-decline, fast-decline, infrequent, continued use, and single-time visitors. These findings contextualize previously reported dichotomous retention rates and provide insights into how patients may utilize the mobile unit to accomplish individualized treatment goals. Future research should incorporate behavioral characteristics and treatment received elsewhere to contextualize how follow-up patterns at a mobile unit relate to MOUD adherence.

## Introduction/Background

Despite the approval and availability of medications to treat opioid use disorder (MOUD), 83% of individuals aged 12 years or older with opioid use disorder (OUD) did not receive MOUD in 2024.(1) Barriers to accessing MOUD include patient-level barriers (e.g., ability to seek care), healthcare system barriers (e.g., insurance), and social barriers (e.g., stigma), which are magnified among individuals who are less willing to interact with the healthcare system and those with unstable housing or low income. (2–5) Low-threshold approaches aim to increase access to OUD treatment by directly addressing barriers through the principles of same-day treatment entry, flexible treatment protocols, individualized treatment goals emphasizing harm reduction, and non-traditional/non-office-based settings.(6–8) An emerging low-threshold model for providing OUD treatment is mobile units that deliver care to individuals within their communities.(8–10)

The University of Illinois Chicago (UIC) Community Outreach Intervention Project (COIP) established a low-threshold mobile medical unit that provides buprenorphine, OUD treatment, and primary care in Chicago neighborhoods with high opioid overdose rates.(11–13) The mobile unit reduced pharmacy-related barriers to accessing treatment by carrying and dispensing buprenorphine on-site at point of care, in contrast to most existing mobile units, which offered buprenorphine prescriptions to be filled at a community pharmacy. The mobile unit was staffed by a specialized physician, a pharmacist who dispensed buprenorphine, an outreach coordinator, a driver, a security guard, and sometimes medical trainees. The renovated recreational vehicle was outfitted to provide patient care, featuring a private examination room and bathroom. In addition to OUD services, patients can visit the mobile unit for primary care services, vitals measurements, chronic disease medication refills, and wound care.

Across the United States, mobile units provide OUD care to at-risk individuals who otherwise may not engage with the healthcare system.(8, 9) Prior research examining the utilization of mobile units for OUD treatment is limited to retention studies reporting metrics such as: rate of treatment retention at various time points, length of engagement (in months), percentage of individuals with lapse in care, and likelihood of returning based on demographic and substance use characteristics.(10) Lacking in the literature is a description of mobile unit follow-up patterns over a longer time frame (i.e., one year), to illustrate how patients utilize the mobile unit for treatment and to contextualize reported retention metrics. Understanding patient follow-up patterns at a mobile unit providing OUD care is useful for organizations operating or establishing a mobile unit to detect utilization trends, assess reach, inform clinical operations to match patient needs, and support budget allocation decisions. This analysis aimed to identify common trajectories, or patterns, of patient follow-up and preliminarily determine patient and system factors that affect follow-up at a mobile unit dispensing buprenorphine and providing OUD treatment.

## Methods

### Data Source and Sample

The UIC COIP mobile unit staff maintained a dataset for every patient encounter since the inception of the mobile unit (May 2021). Clinical encounter details and patient information were captured in a REDCap form by a clinician on the mobile unit, cross-referencing the electronic medical record. A clinician also validated the data quarterly. This analysis included individuals who visited the mobile unit for OUD treatment and/or MOUD from July 29, 2022, when Drug Enforcement Administration (DEA) registration was completed, and buprenorphine was stocked onsite, to September 2025. Additionally, since some individuals visit the mobile unit for other primary care services without receiving OUD treatment, we only included encounters with MOUD as a reason for the visit. Exclusions included individuals under the age of 18 and those with notes suspecting buprenorphine diversion. The dataset was de-identified for this analysis, and, to maintain confidentiality, all results with ≤ 5 individuals were suppressed. The UIC Institutional Review Board approved this study.

### Analysis and Variables of Interest

Group-based trajectory modeling (GBTM) was used to identify common patterns of follow-up observed at the mobile unit. Compared to other trend or growth curve modeling approaches, such as hierarchical modeling and latent curve analysis, which estimate a single average growth/decline trend, the GBTM approach identifies multiple trends and how they evolve over time.(14) Examining utilization with simple average trends over time provides a limited understanding of patterns of use, meanwhile, the GBTM approach examines multiple common trajectories or “types” of use over time to detect meaningful heterogeneous utilization patterns. Although only the frequency of utilization informed the analysis, the underlying assumption of GBTM is that observed patterns reflect the underlying attributes of the individuals who demonstrate them.(14) The SAS 9.4 PROC TRAJ function was used to execute the analysis.(15)

The primary independent variable in the GBTM model was visit frequency over one year following the index encounter. Visit frequency was defined as a binary measure of having at least one mobile unit visit per week; since the UIC mobile unit supplied buprenorphine in 2-, 3-, or 7-day dose packs per visit, individuals would be expected to follow up at least weekly. For most individuals, the index encounter was the date of their first visit to the mobile unit. However, given the mobile unit’s positioning in their neighborhood, individuals may initially visit the mobile unit to learn about services offered but are not yet ready to begin OUD treatment; therefore, an individual’s first encounter may not represent the beginning of their treatment. To account for this, if an individual had a gap in care between their first and second visit greater than one year, we considered the second visit to be their index encounter. For each individual, the 12-month period following their index encounter was included in the analysis, but all subsequent visits beyond this period were excluded to ensure each individual was represented only once in the dataset.

The dependent variable in the GBTM model was the pattern of follow-up at the mobile unit. Those who visited the mobile unit only once were grouped together. The remaining individuals with at least one additional visit after the index encounter were entered in the GBTM to determine their group. Based on the frequency and timing of mobile unit visits since the index encounter, patterns were defined by the likelihood of a mobile unit visit over time, and similar patterns were grouped.

Identifying the best-fitting GBTM involved selecting the optimal number of groups and trajectory shapes (i.e., linear, quadratic, or cubic) to describe the follow-up patterns.(14, 16–18) To ensure statistically meaningful groups, each trajectory group was required to include at least 5% of the sample.(16) The metrics used to assess the fit of the GBTM were the Bayes Information Criterion (BIC), the average posterior probability (AvePP), and the odds of correct classification (OCC).(17) The BIC quantifies how well the model fits the data and allows comparisons between models, with lower values indicating a better fit.(17) AvePP is a measure of internal reliability for each trajectory, and a value above 0.7 suggests similar patterns are grouped within a trajectory, while non-similar patterns are excluded.(17, 18) The OCC measures the odds that an individual was correctly classified into a trajectory group; a value greater than or equal to 5 indicates that the classification was unlikely due to random chance.(18) The significance of the trajectory parameters was also assessed at the α = 0.05 level.

A multinomial logistic regression model identified significant predictors of group assignment by demographic, substance use, and treatment characteristics. Backward elimination, with α = 0.05 as the significance retention criterion, was used to identify significant predictors, and variables with small sample sizes were removed to avoid instability in the estimates. Demographic variables were age (calculated as age at index encounter), sex, ethnicity, and race. Substance use history characteristics included length of substance use (in years as reported at the index encounter), amount of substance used, and history of intravenous drug use (IVDU). The amount and frequency of substance used per day was reported as bags of heroin per day; assuming a ‘bag’ was equivalent to $10 or 0.1g of heroin, recognizing that the contents are not purely heroin. Treatment characteristics included the number of visits for OUD-related treatment, whether buprenorphine was dispensed onsite, and whether individuals were seeking additional services beyond OUD care at their mobile unit visit.

## Results

### Overall Results

**Figure 1:**
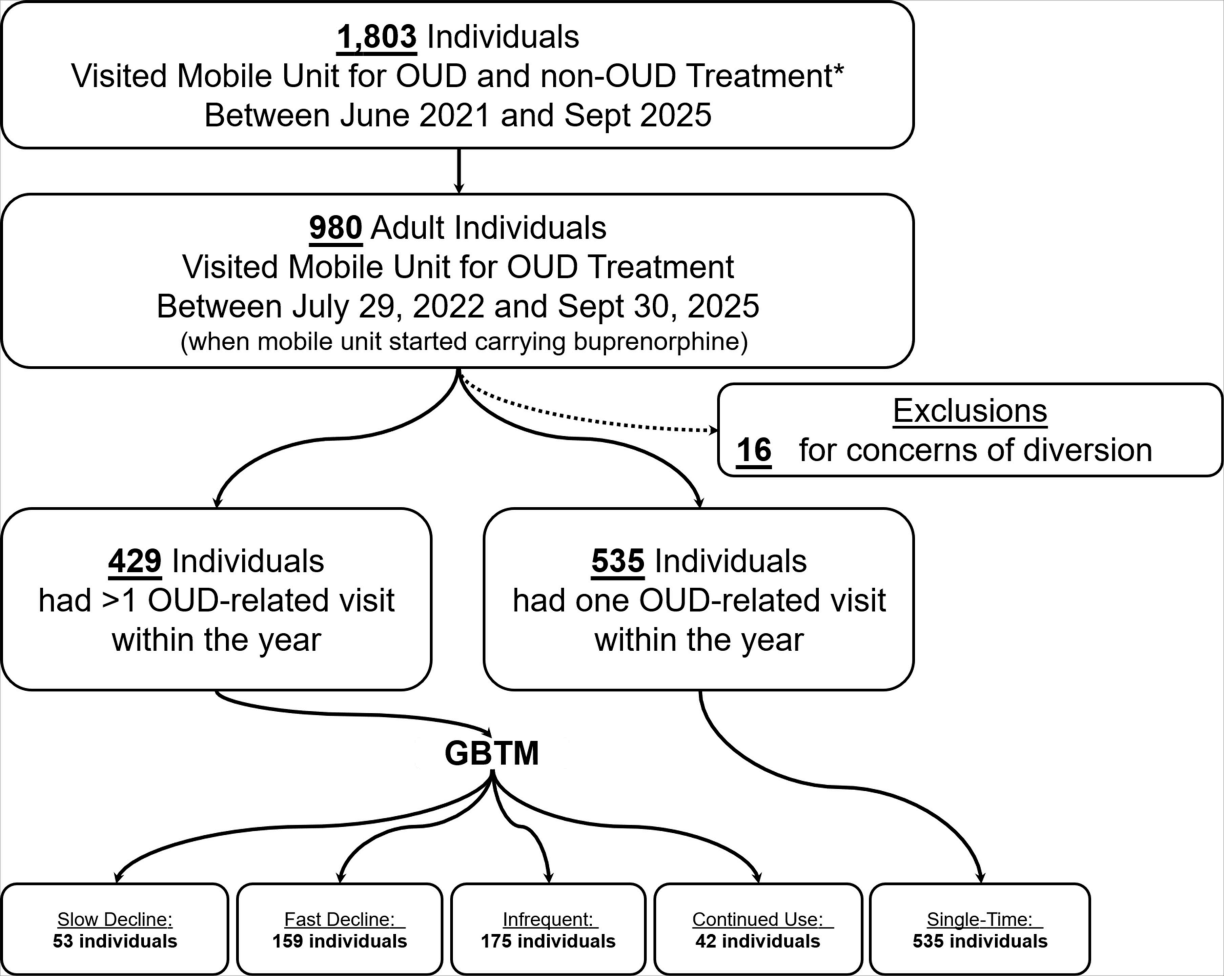
Cohort Flow Diagram *The UIC mobile medical unit offers wound care, vital measurement, and other primary care services to the community OUD: opioid use disorder

From July 29, 2022 to October 31, 2025, when the UIC mobile unit carried buprenorphine onsite, there were 3,528 encounters related to OUD care for 980 individuals. We excluded 16 individuals who were suspected of buprenorphine diversion, based on physician notes. The final patient cohort included in the analysis comprised 964 individuals, of whom 535 had one visit to the mobile unit during the one-year period (Fig. 1).

Characteristics of the individuals are presented in Table 1. The overall cohort had a mean age of 46.4 years, was 72% male, 83.7% had insurance, 12% identified as Hispanic, 58% identified as Black or African American, and 26% identified as White. The mean length of substance use was 19.8 years, and the mean amount used was 5.3 bags per day. The primary route of use was insufflation, and 34% of individuals reported a history of IVDU. Among the overall cohort (n = 964), 44% returned for at least one follow-up visit at the mobile unit, and the mean number of visits for OUD treatment was 3.5 visits. Buprenorphine was dispensed on-site in 51% of encounters, and patients were seeking additional services beyond OUD care in 23% of encounters.

### Group-Base Trajectory Model Results

The GBTM was run among the 429 individuals who had more than one visit to the mobile unit, and the four-group model with linear coefficients was the best-fitting model. All group memberships had at least 5% of the sample; when testing a five-group model, at least one group had a membership of less than 5% of the sample. A four-group model also had a higher BIC compared to a three-group model, and the resulting groups in the four-group model were more meaningful to our research question. When testing the best-fitting coefficients for the model parameters, the linear coefficients had the lowest BIC. The four-group model with linear coefficients also had an AvePP above 0.85 for each trajectory, and the OCC was greater than 5 for each trajectory. Lastly, all linear coefficients were significant at α = 0.05.

**Figure 2:**
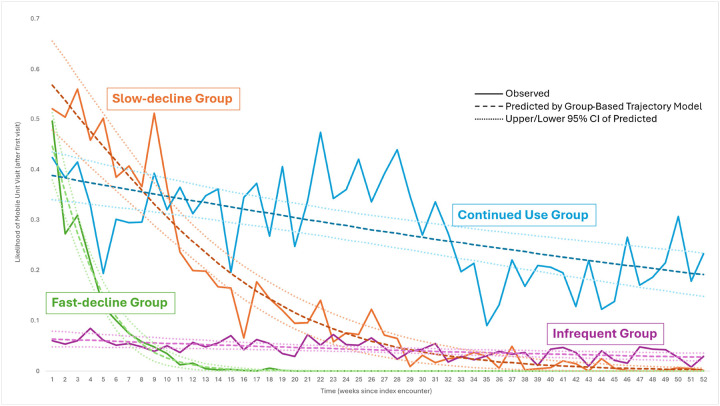
Patterns of Follow-up Among Those with >1 Visit

The group of individuals who visited the mobile unit once in the one-year observation period was added to the four GBTM groups to yield five total groups of mobile unit utilizers: slow-decline (n, %: 53, 6%), fast-decline (159, 16%), infrequent (175, 18%), continued use (42, 4%), and single-visit (535, 56%). The trajectories of groups with more than one visit to the mobile unit are shown in Fig. 2. Group descriptions are presented in Table 1. The average number of visits for OUD treatment over the year post-index encounter was 9.6 in the slow-decline group, 3.1 in the fast-decline group, 4.9 in the infrequent group, and 22.6 in the continued use group. The predicted likelihood of a visit ranged from 57% to 0%, with a gradual decrease over 30 weeks post-index encounter, in the slow-decline group, and 45% to 0%, with a decrease over 10 weeks, in the fast-decline group. The predicted likelihood of a visit ranged between 6% and 3% in the infrequent group, and 39% and 19% in the continued use group over the year post-index encounter.

### Characteristics of Identified Groups

Demographic, treatment, and substance use characteristics of each group are presented in Table 1. The full multinomial logistic regression model included age, sex, race, insurance, buprenorphine dispensed, historical amount used, length of substance use, and history of IVDU variables; ethnicity was removed due to small estimate sizes, and historical routes of use were removed as they were directly related to history of IVDU. In backward elimination, no covariates met retention criteria, indicating none of the predictors were significantly associated with group assignment. Table 2 presents odds ratios and 95% confidence intervals from the full model.

Demographics were relatively evenly distributed, with continued use (44.5 years, 64% male) and infrequent (45.2 years, 67% male) groups having a slightly younger age and slightly more females, on average, than the slow-decline (46.3 years, 74% male) and fast-decline groups (46.7 years, 72% male). Compared with single-time visitors, males were significantly less likely to fall in the infrequent group (p < 0.05). The slow-decline group had the highest proportion with insurance (92%), while single-time visitors had the lowest (78%). Compared with single-time visitors, slow-decline, infrequent, and continued use visitors were significantly more likely to have insurance (p < 0.05). Race and ethnicity were also distributed relatively evenly across the groups, with continued use and fast-decline groups having higher proportions of individuals identifying as Black or African American (69% and 64%, respectively), and slow-decline group having the highest proportion of individuals identifying as White (38%). Compared to single-time visitors, individuals identifying as Black or African American were significantly more likely to be in the continued use and fast-decline groups (p < 0.05).

In substance use history characteristics, continued use visitors had the shortest average length of substance use history (17.6 years) and the lowest average amount of substance used (4.3 bags). Among all groups, insufflation was the most common route. Slow-decline visitors had the highest percentage of individuals with a history of IVDU.

Treatment characteristics among the five groups included a predicted likelihood of having a visit four weeks after the index encounter of 48% for the slow-decline group, 21% for fast-decline, 6% for infrequent, and 37% for continued use. The slow-decline group had the highest proportion of visits where buprenorphine was dispensed on-site at the mobile unit (57%). Compared with single-time visitors, those in the fast-decline and infrequent groups were significantly less likely to receive buprenorphine at their mobile unit visit (p < 0.05). Lastly, the infrequent group had the highest number of visits seeking other services in addition to OUD treatment (28%).

## Discussion

This study used a dataset spanning 38 months and including over 900 patients to characterize follow-up patterns at a mobile unit that provided OUD treatment and buprenorphine on-site to neighborhoods with unmet needs. Our findings highlight the complexities of patient follow-up patterns at mobile units providing OUD treatment, which are not conveyed with dichotomous retention outcomes as previously reported.(10) Documenting utilization patterns informs economic planning and provides insights into how patients may perceive the mobile unit as part of their treatment plan.

Previous literature characterizes utilization of mobile units providing OUD treatment by dichotomous retention metrics.(10) The Mobile Overdose Response Unit (Philadelphia, PA) reported that of 237 patients 86% returned for at least one follow-up visit, and 69% completed four or more visits.(19) The Begin the Turn mobile unit (Philadelphia, PA) reported one, three, and five-month retention rates of 61.2% (90/147), 36.6% (30/82), and 27.6% (8/29), respectively, among 147 individuals.(20) The Project Connections at Re-Entry (PCARE) van (Baltimore, MD) reported that of 190 patients, 67.9% returned for at least one additional visit, and 31.6% were engaged in treatment 30 days after the initial visit.(21) In a later study of the PCARE among 566 patients, 93.8% were engaged in treatment for at least 30 days, and 80% for at least 90 days.(22) And, the Healthcare on the Spot mobile clinic (Baltimore, MD) reported that of 420 patients, 56.0% were still receiving care at one month and 26.9% at three months.(23) These observed retention rates in other mobile unit programs were higher than the rates observed in our model for any group, which may be attributed to differences in patient screening, treatment entry requirements, and patient populations.

Several factors of the UIC mobile unit, including the program aims, transient patient population, and differing patient preferences, contribute to the observed retention rates and utilization patterns. While many OUD mobile units aim to engage patients in treatment with the eventual goal of connecting them to other long-term treatment programs, the UIC COIP program can serve as a primary source of treatment, thereby engaging a larger patient population and capturing broader patient follow-up behavior. Furthermore, a substantial proportion of patients at the UIC mobile unit are transient, either moving between cities/states or going in and out of the criminal justice system, which may explain gaps in treatment.(12, 13) Furthermore, some patients report the mobile unit location as triggering or find aspects of the mobile unit operation incompatible with their preferences, and they choose to transition to care elsewhere.(24)

Our results can be used to expand on the dichotomous retention metrics reported in other studies, providing insight into how individuals utilize a mobile unit for OUD treatment over time. For example, in our sample, the continued use visitors had the highest average number of visits in the year, but a predicted likelihood of having a visit ranging from 19% to 39% over the year after the index encounter. Looking only at the dichotomous retention rates (ranging from 19% to 39%) in this group would not convey the consistency of engagement with the mobile unit over time.

Our identified utilization patterns may allude to how individuals perceive the mobile unit in their treatment plan. While we did not have data to characterize whether patients sought or received OUD care elsewhere after visiting the mobile unit, qualitative research conducted with mobile unit patients supports that the mobile unit served different roles, ranging from being the primary source of MOUD, being an as-needed resource, or being an entry-way to treatment (providing a low-commitment trial prior to entering a treatment program).(24) Based on patient perspectives of the mobile unit(24) and UIC COIP provider insights, the slow-decline visitors likely used the mobile unit as their primary source of MOUD, and either transferred to another long-term program for continued treatment, concluded treatment, or returned to use after stopping visits to the mobile unit. The fast-decline visitors may have used the mobile unit as an accessible introduction to treatment, but likely either transferred to an alternative treatment program after deciding the mobile unit was not compatible with their medication/treatment preferences, or decided they were not ready for treatment. The infrequent visitors likely used the mobile unit as an as-needed service when they either could not get MOUD from their primary source or after a transient return to use (one-time ingestion) to have buprenorphine on hand to prevent additional cravings. Continued use visitors could be taking a harm-reduction approach to treatment and tailoring buprenorphine use to individualized treatment goals (which align with low-threshold treatment principles) and/or using the mobile unit as an as-needed treatment option. Additional investigations of how patient perceptions, behavioral factors, and internal motivations for treatment correlate to mobile unit utilization patterns are necessary to validate these conjectures.

### Limitations

While our results provide valuable insights into mobile unit utilization, it is worth acknowledging the limitations in our dataset and approach. First and most significantly, we did not have access to data on OUD care or buprenorphine received elsewhere while patients were visiting the mobile unit, or after they stopped visiting the mobile unit. Because tracking each individual to capture where all they received treatment during and after their mobile unit visit was not feasible, we were unable to draw conclusions on how the observed follow-up patterns related to individuals' continued engagement in MOUD treatment. Additional data on behavioral characteristics that influence an individual’s decision to adhere to treatment (such as perceived need for treatment and past treatment experiences), coupled with data on OUD treatment external to the mobile unit, may help explain differences between patients currently assigned to the single-visit and fast-decline groups.

Secondly, the dataset was not linked to the patient’s medical records, including those of the UIC medical center, with which COIP was affiliated. We included all relevant clinical variables present in the data, but acknowledge that additional variables, especially related to overdoses or hospitalization events, detailed substance use history, comorbidities, and utilization of general healthcare services, would enhance our findings. Third, as with all secondary data analyses, the dataset in this study was not created specifically for this analysis and may have missingness, inconsistencies, and bias. Since the dataset was maintained by clinicians who also staffed the mobile unit, the data were relatively complete and periodically checked for internal consistency, but inconsistencies may persist due to changes in the data collection form over time, variations in how different clinicians entered and validated the data, and potential reporting/memory biases. Lastly, this study was conducted with patients in Chicago, Illinois, which has unique characteristics in OUD prevalence, the mobile unit program structure, and city-level healthcare access characteristics. Therefore, generalizations of our results to other regions and mobile unit structures should consider differences in the populations served.

## Conclusions and Future Directions

This research used group-based trajectory modeling to identify five common patterns of follow-up at a mobile unit offering OUD treatment in an urban setting. Characterizing the common patterns of follow-up provided context for commonly reported retention metrics and illuminated how individuals may incorporate the low-threshold mobile unit into their personal treatment plans. Future research should focus on whether patients remain in treatment after engaging with the mobile unit and on characterizing individuals who are more likely to remain engaged in treatment, both during and after their interaction with the mobile unit.

## Figures and Tables

**Table 1:** Characteristics of Patients Overall and by Group

Description	Overall	Slow-Decline	Fast-Decline	Infrequent	Continued Use	Single-Time
		Utilized the mobile unit regularly for multiple months but eventually stopped and did not restart for the rest of the year	Utilized the mobile unit regularly for about one month, then stopped and did not restart for the rest of the year	Utilized the mobile unit infrequently over the course of the year	Utilized the mobile unit multiple times over the year, with an on & off pattern	Utilized the mobile unit once and did not return for the rest of the year
Demographic Characteristics						
N (%)	964 (100%)	53 (6%)	159 (16%)	175 (18%)	42 (4%)	535 (56%)
Mean Age [95% CI]	46.4 [45.7,47.1]	46.3 [43.2,49.3]	46.7 [45,48.3]	45.2 [43.4,46.9]	44.5 [40.7,48.2]	46.9 [45.9,47.9]
Sex (n, % male)	698 (72%)	39 (74%)	115 (72%)	118 (67%)	27 (64%)	399 (75%)
Insurance (% Insured)	83.7%	91.9%	88.7%	82.2%	88.2%	77.9%
Ethnicity (n, % Hispanic)	112 (12%)	x	11 (7%)	19 (11%)	x	74 (14%)
Race (n, %)						
Black or African American	559 (58%)	30 (57%)	101 (64%)	100 (57%)	29 (69%)	299 (56%)
White	247 (26%)	20 (38%)	35 (22%)	50 (29%)	8 (19%)	134 (25%)
Other	100 (9%)	x	10 (7%)	17 (10%)	x	69 (13%)
Unknown	58 (6%)	x	13 (8%)	8 (5%)	x	33 (6%)
Mean Length of Substance Use (years)	19.8 [18.8,20.7]	21.2 [17.1,25.3]	19.6 [17.3,21.8]	18.5 [16.5,20.6]	17.6 [13.3,21.9]	20.4 [19.1,21.8]
Mean Amount Used (# of bags) [95% CI]	5.3 [5,5.6]	5.4 [4.1,6.6]	5.2 [4.4,6]	5 [4.5,5.5]	4.3 [3.4,5.2]	5.6 [5.2,6.1]
IVDU History (% Yes)	289 (34%)	22 (42%)	43 (30%)	57 (34%)	14 (35%)	153 (34%)
Route (n, %)						
Insufflation Only	547 (64%)	29 (56%)	98 (68%)	111 (66%)	22 (55%)	287 (64%)
Injection Only	70 (8%)	7 (13%)	11 (8%)	10 (6%)	x	40 (9%)
Oral Only	17 (2%)	x	x	x	x	8 (2%)
Multiple Routes	219 (26%)	15 (29%)	32 (22%)	47 (28%)	12 (30%)	113 (25%)
Mobile Unit Treatment Characteristics						
Predicted Likelihood of Visit 4 weeks after index encounter		48%	21%	6%	37%	
Mean Visits for OUD Treatment [95% CI]	3.5 [3.1,3.9]	9.6 [8.7,10.5]	3.1 [2.9,3.3]	4.9 [4,5.7]	22.6 [19.2,26]	1 [1,1]
Buprenorphine Dispensed on-site at Visit (%)	51%	57%	54.5%	54.9%	44%	49.7%
Seeking Additional Services Beyond OUD Care (i.e., wound care, vitals)	23%	23%	21%	28%	21%	22%

**Table 2:** Multinomial Logistic Regression Results of Characteristics Among Groups

Predictor	Interpretation/Reference Value	Odds Ratio and 95% Confidence Interval by Group (reference: Single-Time Group)			
		Slow-Decline	Fast-Decline	Infrequent	Continued Use
Age	One year increase in age	0.99 [0.95, 1.03]	1.01 [0.98, 1.03]	0.99 [0.96, 1.01]	0.97 [0.92, 1.01]
Sex	Male (reference: Female)	0.94 [0.42, 2.11]	0.76 [0.44, 1.33]	0.51 [0.31, 0.82]*	0.55 [0.24, 1.26]
Race	Black or African American (reference: Other/Unknown)	3.17 [0.88, 11.48]	2.56 [1.22, 5.36]*	1.77 [0.95, 3.29]	4.33 [1.19, 15.71]*
	White (reference: Other/Unknown)	3.58 [0.97, 13.22]	1.23 [0.52, 2.88]	1.36 [0.69, 2.68]	0.84 [0.18, 4.03]
Insurance	Having insurance (reference: not having insurance)	4.8 [1.42, 16.19]*	1.75 [0.96, 3.19]	3.23 [1.75, 5.98]*	13.18 [1.74, 100.1]*
Buprenorphine Dispensed	Receiving buprenorphine (reference: not receiving buprenorphine)	0.91 [0.47, 1.75]	0.57 [0.36, 0.91]*	0.65 [0.42, 0.99]*	0.93 [0.44, 1.97]
Mean Amount Used (# of bags)	One bag increase in amount	0.95 [0.86, 1.05]	0.99 [0.93, 1.05]	0.98 [0.92, 1.03]	0.91 [0.8, 1.03]
Length of Substance	One year increase in length of use	1.01 [0.97, 1.04]	0.98 [0.96, 1.01]	0.99 [0.97, 1.01]	1 [0.96, 1.03]
IVDU History	History of IVDU (reference: no history of IVDU)	1.5 [0.7, 3.23]	0.96 [0.55, 1.69]	0.94 [0.57, 1.54]	1.38 [0.59, 3.24]

* The UIC mobile medical unit offers wound care, vital measurement, and other primary care services to the community

OUD: opioid use disorder

## Data Availability

The datasets analyzed during the current study are not publicly available due patient privacy reasons. Please contact the corresponding author for additional information or to connect with the UIC COIP team for data requests.

## Abbreviations

AvePP: Average Posterior Probability
BIC: Bayes Information Criterion
COIP: Community Outreach Intervention Project
DEA: Drug Enforcement Administration
GBTM: Group-based trajectory modeling
IVDU: Intravenous Drug Use
MOUD: Medications for Opioid Use Disorder
OCC: Odds of Correct Classification
OUD: Opioid Use Disorder
PHI: Protected Health Information
UIC: University of Illinois Chicago
